# Supplementary material for: Early Cretaceous Keteleerioxylon Wood in the Songliao Basin, Northeast China, and Its Geographic and Environmental Implications
Source: Biology (Basel). 2022 Nov 7;11(11):1624. doi: 10.3390/biology11111624 (PMC9687590; doi:10.3390/biology11111624)
Supplement: Supplementary file 1 [file biology-11-01624-s001.zip › Table S1-shi.pdf]

Table S1. Comparison of wood anatomical characters of *Keteleerioxylon changchunense* sp. nov. and closely related fossil species (?, no data)

Anatomical characters	Pits on radial walls of tracheids	Diameter of pits (μm)	Pits on tangential walls of tracheids	Crassula	Height of rays (in cells)	Number of biseriate layer	Marginal ray cells of the ray tracheid type	Transverse walls of axial parenchyma	Number of epithelial cells in vertical resin canals	Number of pits in the cross-field	Diameter of cross-field pits (μm)	Type of pitting
***Keteleerioxylon changchunense* sp. nov.**	**Uniseriate to triseriate**	**(11)12–17(20)**	**absent**	**Present**	**(1)6–11(37)**	**1–8**	**Present**	**Smooth**	**6-11**	**1–3(6)**	**5-10**	**Taxodioid and cupressoid**
Keteleerioxylon arcticum Shilkina [7]	Uniseriate and biseriate	?	uncommon	Present	1–24	Uncommon (rarely triaseriate)	Absent	Smooth	?	1–3(4)	?	Taxodioid
Keteleerioxylon fokinii Shilkina [34]	Uniseriate and biseriate	?	Absent	Absent	1–30	Uncommon	?	Smooth	?	1–2(3)	?	Cupressoid
Keteleerioxylon primoryense Blokh. [35]	Uniseriate and biseriate	12-18	uncommon	Present	1-40	1–4 (10)	Present	Smooth	(5)6–8(10)	1–3(5)	6-7.5	Taxodioid
Keteleerioxylon kamtschatkiense Blokh. et Afonin [11]	Uniseriate and biseriate	(12)18-24(27)	Present	Absent	1-30 (46)	1–3	Present	Smooth	6–10	1–3(5)	5	Taxodioid and cupressoid
Keteleeria mabetiensis Watari [37, 38]	Uniseriate to triseriate	?	Present	Present	1-36	1–4	Present	Knotty	?	1–4(5)	?	Taxodioid and piceoid
Keteleeria zhilinii Blokh. et Bondarenko [10]	Uniseriate and biseriate	(10)12-18(24)	Present	Uncommon	1-25	1–3(4)	Present	2-3 knots	(5)6–7(8)	1–4(5)	6-9	Taxodioid
"Keteleeria fortunei" (Andr. Murray) Carr. [39]	Uniseriate and biseriate	15-19	?	?	(2)10-30(40)	Uncommon	?	Axial parenchyma absent	4-6	1-4	?	Taxodioid
Keteleeria sp. 1 [40]	Uniseriate and biseriate	17-22	?	Present	(1)7-12(24)	Uncommon	?	Knotty	5-8	1-4(commonly 2)	?	Taxodioid
Keteleeria sp. 2 [12]	Uniseriate and biseriate	12-22	?	Present	1-18	?	?	Knotty	3-14	1-2(4)	5-9	Taxodioid and cupressoid
